# In Vitro Evaluation of Vancomycin-Induced Toxicity in Human Primary Knee Chondrocytes

**DOI:** 10.1177/10915818231216413

**Published:** 2023-11-21

**Authors:** Susan Hall, Jane Grayson, Gary Grant, Christopher Vertullo, Shailendra Anoopkumar-Dukie

**Affiliations:** 1 School of Pharmacy and Medical Sciences, 97562 Griffith University, Gold Coast, Australia; 2 School of Health Sciences, 638093 The University of Sydney, Glebe, Australia; 3 The Knee and Sports Medicine Centre, Gold Coast, Australia

**Keywords:** vancomycin, chondrocytes, apoptosis, reactive oxygen species production, surgical infection

## Abstract

Septic arthritis as a complication of orthopaedic joint surgery can have catastrophic outcomes for patients. To minimise infection risk associated with elective orthopaedics, topical vancomycin during surgery has become increasingly common. Evidence suggests that high concentrations of vancomycin, following direct application of the drug to the joint, are toxic towards various local cell types in the joint, including chondrocytes. However, the mechanism of this vancomycin tissue toxicity is yet to be determined. The aim of this study was to evaluate the toxicity of vancomycin on chondrocytes and the mechanisms of cell death involved. Human primary knee chondrocytes were exposed to vancomycin (1.25–10 mg/mL) for 24 h and their viability assessed using the resazurin reduction assay in vitro. Specific cell death mechanisms and their contributors, including reactive oxygen species (ROS) production and apoptosis, were measured. This study showed that high concentrations of vancomycin (5 and 10 mg/mL) were toxic towards human primary knee chondrocyte cells, while lower concentrations (1.25 and 2.5 mg/mL) were not. Cell death studies found that this occurred through an apoptotic pathway. This study provides additional support that vancomycin in high doses is toxic towards chondrocytes and preliminary evidence that this toxicity occurs via apoptotic cell death mechanisms.

## Background

Surgical site infections in elective surgery are associated with morbidity and, although uncommon with an estimated incidence up to 5.4%, the outcomes from these infections have devastating consequences.^1^ This is despite the development of numerous strategies to manage their occurrence including administration of preoperative antibiotics, advances in surgical preparation methods, and wound care measures.^2^ An increasingly common practice shown to reduce the incidence of septic arthritis in orthopaedic surgery is the local application of vancomycin to the surgery site and/or graft.^2^

Vancomycin, a glycopeptide antibacterial agent, has both bacteriostatic and bactericidal activity dependent on the bacterial species that it is being used to treat.^3^ Its use has increased with the emergence of methicillin resistant *Staphylococcus aureus* (MRSA).^4^ In addition to this, the use of local vancomycin in orthopaedic surgery has become common practice to reduce the incidence of joint infection. It is used in a range of surgeries including spinal surgery,^5^ operatively treated bone fractures,^6^ anterior cruciate ligament (ACL) reconstruction,^2^ and total hip and knee arthroplasty.^7,8^

Various methods of topical vancomycin application have been reported and vary according to the type of surgery being performed. Amongst these, the local application of vancomycin powder,^5,9^ pre-soaking of tissue grafts in a vancomycin solution^10^ and loading vancomycin into bone cement,^11^ are clinically popular. In ACL reconstruction specifically, higher infection rates occur with hamstring tendon grafts in comparison to bone-patellar tendon-bone grafts. However, both surgical methods can result in infection and potentially lead to a poor patient outcome.^10^ This has led to the evaluation of using pre-soaked vancomycin hamstring tendon grafts in anterior cruciate ligament reconstruction as a measure to reduce infections associated with this type of surgery.^10^ When compared to intravenous pre-operative antibacterial administration in observational datasets, vancomycin pre-soaked hamstring tendon grafts induced a statistically significant reduction in surgery-related local infections, with an infection rate of 1.4% versus 0%.^10^ Additionally, the use of local vancomycin has been shown to be highly cost effective through decreasing the risk of repeat interventions for infection debridement, length of stay, and prolonged postoperative recovery that occurs after surgical site infection.^9,12^ Although beneficial, the major limiting factor for the use of local vancomycin is potential toxicity to adjacent tissue in the joint.^5,9,13,14^ Direct application of vancomycin to the joint has been shown to expose the various cell types in the joint to high concentrations, greater than 2 mg/mL when used as prophylaxis^15^ and much higher concentrations, on average approximately 10 mg/mL in the treatment of intra articular infections.^16^ However, when tendons are pre-soaked for prophylaxis, it is estimated that chondrocyte exposure of 45 to 62 μg/mL to vancomycin occurs, much lower than after direct joint application.^2^

Previous studies have evaluated and confirmed the toxicity of vancomycin, particularly in high concentrations, to numerous structures in joints including chondrocytes,^5,9^ osteoblasts^5,14^ and skeletal muscle cells.^5^ Dose-dependent toxicity has been observed with local administration of vancomycin, with high doses resulting in the death of osteoblasts^13^ along with inhibiting the differentiation of osteoblasts causing changes to the bone healing process.^14^

To date, there is evidence to suggest that high concentrations of vancomycin are toxic towards chondrocytes. However, investigations into how this occurs mechanistically have yet to be undertaken. Therefore, the aim of the current study was to evaluate the toxic effect of vancomycin exposure towards the viability of primary human knee chondrocytes and investigate the cell-death mechanisms by which this occurred.

## Materials and Methods

### Cell Culture

Human primary chondrocytes (NHAC-Kn) were purchased from Lonza (Basel, Switzerland). Cells were grown and maintained at 37°C and 5% CO_2_ in Chondrocyte Basal Medium (CBM^TM^ Medium) with the contents of a Clonetics™ CGM™ BulletKit™ (Lonza Catalog No. CC-3216) containing R3-Insulin-like Growth Factor-1, human recombinant Fibroblast Growth Factor-Beta (hrFGF-β), transferrin, insulin and 10% Foetal Bovine Serum (FBS). All experiments were performed using an antimicrobial free medium.

### Preparation of Working Solutions

Stock solutions of 50 mg/mL of vancomycin hydrochloride (Sigma Aldrich, St Louis, MO, USA) in sterile phosphate buffered saline (PBS) were prepared and stored at −20°C. All working solutions (1.25 mg/mL, 2.5 mg/mL, 5 mg/mL, 10 mg/mL and appropriate vehicle control) were prepared fresh on the day of experiments using medium. The concentration range of vancomycin used in the current study was chosen to encompass clinically relevant doses when vancomycin is applied directly to the joint.^15,16^ Additionally, with concentrations chosen aligned with those used in previous in vitro studies investigating the toxicity of vancomycin toward chondrocytes to allow for further investigation of potential mechanisms attributed to the observed cytotoxicity.^5,9^

### Resazurin Proliferation Assay

Reduction of the redox dye resazurin to resorufin was used to measure the proliferation of cell cultures.^17,18^ Briefly, chondrocyte cells were seeded at 1 × 10^5^ of trypan blue excluding cells/mL in 24 or 96-well microtitre plates. After 24 h, cells were exposed to various concentrations of vancomycin (1.25, 2.5, 5 and 10 mg/mL) for a further 24 h. Following the appropriate incubation, the culture medium was removed and replaced with fresh medium containing 44 μM of resazurin (Sigma-Aldrich, St Louis). Cultures were then incubated for a further 1 h and subsequently the reduction of resazurin to resorufin was determined using fluorescence (excitation 530 nm; emission 590 nm) using Tecan Infinite M200 Pro (Tecan, Mannedorf, Switzerland).

### 2',7'-Dichlorofluorescein Assay

2',7'-Dichlorodihydrofluorescein diacetate (H_2_DCFDA) is a non-fluorescent chemical used as an indicator for ROS in cells. H_2_DCFDA is permeable to the cell where, upon cleavage of the acetate groups by intracellular esterases and oxidation, is then converted to the highly fluorescent 2',7'-dichlorofluorescein (DCF).^19^ H_2_DCFDA was used to measure global ROS production as previously described.^19^ Briefly, chondrocyte cells were seeded at 1 × 10^5^ of trypan blue excluding cells/mL in 24 or 96-well microtitre plates. After 24 h, cells were exposed to various concentrations of vancomycin (1.25, 2.5, 5 and 10 mg/mL) for a further 24 h. Following incubation for 24 h, medium above the cells was replaced with serum-free medium containing H_2_DCFDA (10 μM) for 30 min. Cells were then washed twice with PBS and fluorescence due to the oxidized dye (excitation 485 nm; emission 535 nm) was measured using a Tecan Infinite M200 Pro (Tecan, Mannedorf, Switzerland).

### Caspase-3 Activation

Caspase-3 activation was used in the current study as a measure of early apoptosis. Cells were seeded at a density of 1 × 10^5^ cells/mL, incubated for 24 h and then treated as described earlier. Caspase-3 activity was determined using a caspase-3 fluorescence assay kit (Cayman Chemicals, Michigan, USA). All steps were performed according to the manufacturer’s instructions.

### JC-1 Mitochondrial Potential Assay

The JC-1 mitochondrial potential assay was used to assess cellular mitochondrial membrane stability as a secondary measure of apoptosis. Cells were seeded at 1 × 10^5^ cells per mL in 24-well plates and treated at 24 h with various concentrations of vancomycin (1.25, 2.5, 5 and 10 mg/mL). At 48 h, media was gently aspirated, and wells washed twice with PBS. Under subdued lighting, 500 μL JC-1 dye (1 μg/mL in complete media) was added to wells for 30 min, after which wells were washed twice using PBS. Subsequently, 500 μL PBS was added to each well and red and green fluorescence read using a Tecan Infinite M200 Pro (Tecan, Mannedorf, Switzerland) (red - excitation: 530 nm, emission: 590 nm; green - excitation: 485 nm, emission: 535 nm).

### Crystal Violet Staining

Crystal violet staining of the treated cells was done to assess morphological changes in response to treatment. Briefly, cells were placed on ice and washed 2X with cold PBS. Cells were then fixed on ice for 10 min with ice-cold methanol. Methanol was then aspirated, and the cells washed 3X with water. Cells were then exposed to .5% Crystal Violet solution in 25% methanol and left to incubate for 10 min at room temperature. The crystal violet solution was then aspirated, and the cells washed 3X with water and allowed to dry at room temperature overnight and visualised using Olympus IX53 microscope (Olympus, Victoria, Australia).

### Statistical Analysis

All results were represented as a mean ± standard deviation of a minimum of three replicates of three independent experiments. One-way ANOVA with Tukey’s post hoc test (if significance observed) were used in this study and were performed using GraphPad InStat version 3.06 (2003) with *P* < .05 (*), *P* < .01 (**), *P* < .001 (***) and *P* < .0001 (****). All graphs were drawn using GraphPad Prism v6 (San Diego, USA).

## Results

### Effects of Vancomycin on the Viability of Human Primary Chondrocyte Cells

**Figure 1.**
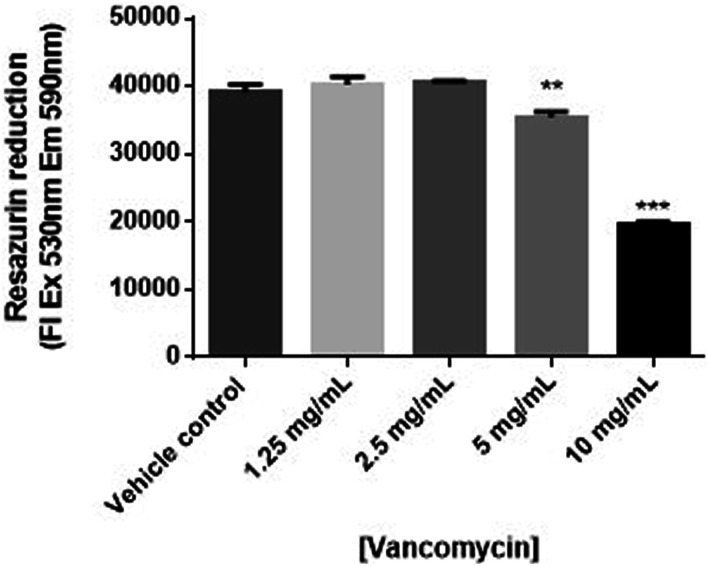
Changes in viability measured by resazurin reduction in human primary chondrocytes exposed to vancomycin 1.25 to 10 mg/mL relative to vehicle control (n = 3), where *P* < .05 (*), *P* < .01 (**) and *P* < .001 (***).

Resazurin reduction to resorufin was used to measure the cytotoxic effects of vancomycin in human primary chondrocyte cells. As shown in Figure 1, vancomycin (5 and 10 mg/mL) significantly decreased the viability of human primary chondrocyte cells in a concentration-dependent manner, when compared to vehicle control (*P* < .05 and .0001, respectively). Exposure of chondrocytes to 10 mg/mL of vancomycin resulted in a reduction in viability by approximately 50%.

### Effects of Vancomycin on Reactive Oxygen Species Production in Human Primary Chondrocyte Cells

**Figure 2.**
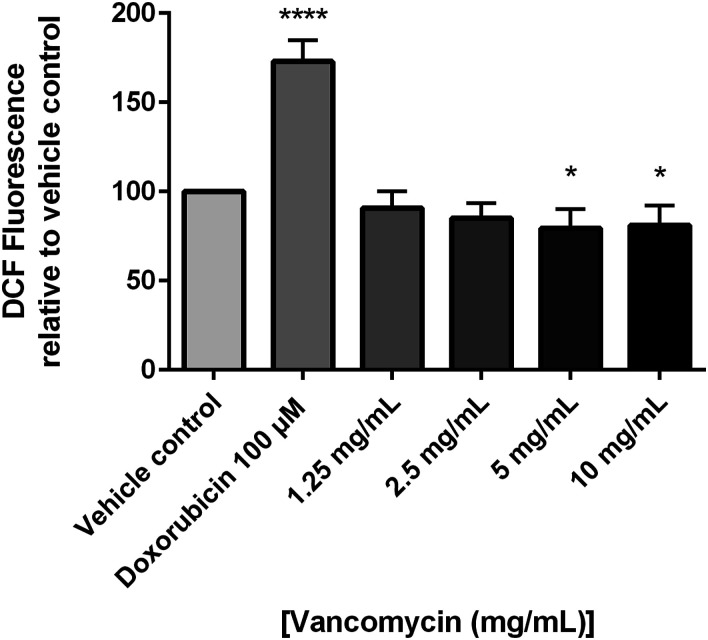
Changes in reactive oxygen species generation measured by DCF fluorescence in human primary chondrocytes exposed to vancomycin 1.25 to 10 mg/mL relative to vehicle control and positive control, doxorubicin (n = 3), where *P* < .05 (*), *P* < .01 (**) and *P* < .001 (***).

To determine if the cytotoxic effects of vancomycin were due to effects on free radical production, chondrocytes were treated with vancomycin and free radical production was measured using DCFH-DA. Overall free radical production, as measured by DCFH-DA fluorescence relative to cell number, was found to be decreased in vancomycin (5 and 10 mg/mL) treated chondrocytes in comparison to vehicle control (*P* < .05) (Figure 2).

### Effects of Vancomycin on Chondrocyte Apoptosis

**Figure 3.**
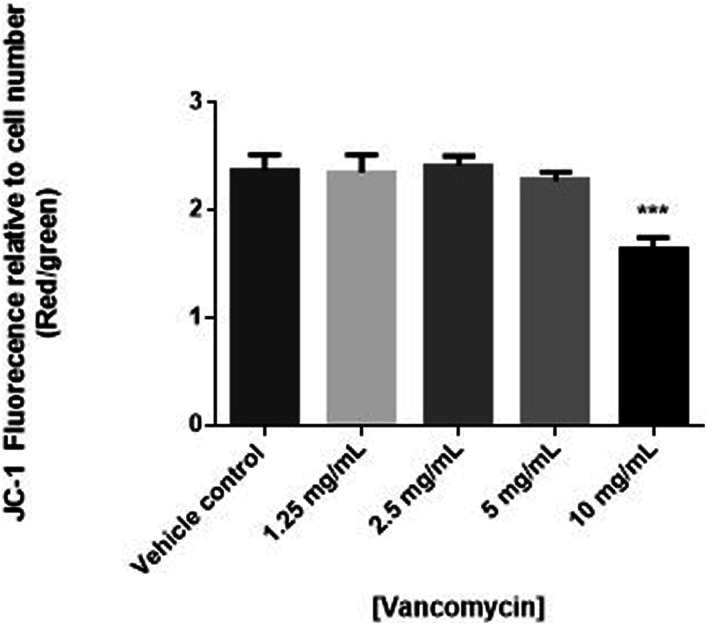
Changes in apoptosis measured by changes in mitochondrial membrane potential (n = 3) in human primary chondrocytes exposed to vancomycin 1.25 to 10 mg/mL relative to vehicle control, where *P* < .05 (*), *P* < .01 (**) and *P* < .001 (***).

**Figure 4.**
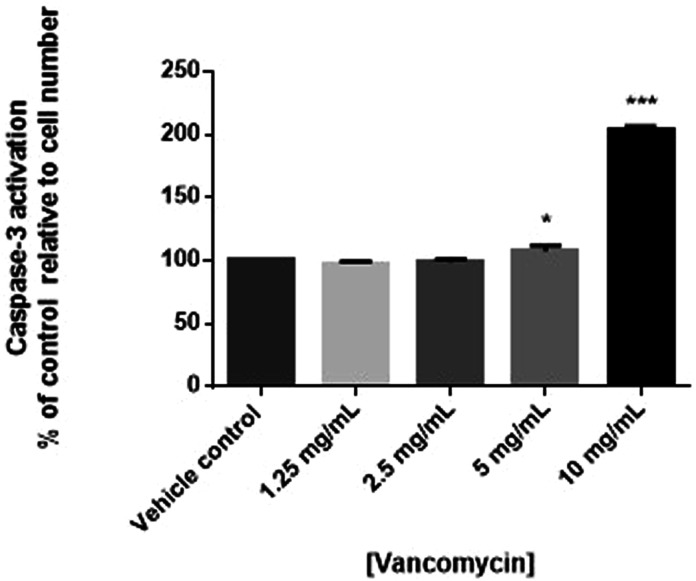
Changes in apoptosis measured by changes caspase-3 activation (n = 3) in human primary chondrocytes exposed to vancomycin 1.25 to 10 mg/mL relative to vehicle control, where *P* < .05 (*), *P* < .01 (**) and *P* < .001 (***).

To determine if the cytotoxic effects of vancomycin were due to effects on apoptosis, human chondrocyte cells were treated with vancomycin and mitochondrial membrane potential measured using JC-1. Consistent with viability results, statistically significant decreases in mitochondrial potential, suggestive of apoptosis, were observed in human primary chondrocytes exposed to 10 mg/mL of vancomycin (*P* < .0001). Although not statistically significant, a trending decrease in mitochondrial potential was observed in cells exposed to 5 mg/mL vancomycin (Figure 3). To confirm apoptosis was occurring, the expression of caspase-3 measured. Consistent with JC-1 results, decreased cell viability was associated with increased activation of caspase-3, further suggesting early apoptosis, when human primary chondrocytes were exposed to vancomycin (5 and 10 mg/mL) (*P* < .05 and .001, respectively) (Figure 4).

### Effect of Vancomycin of the Morphology of Human Primary Chondrocytes

**Figure 5.**
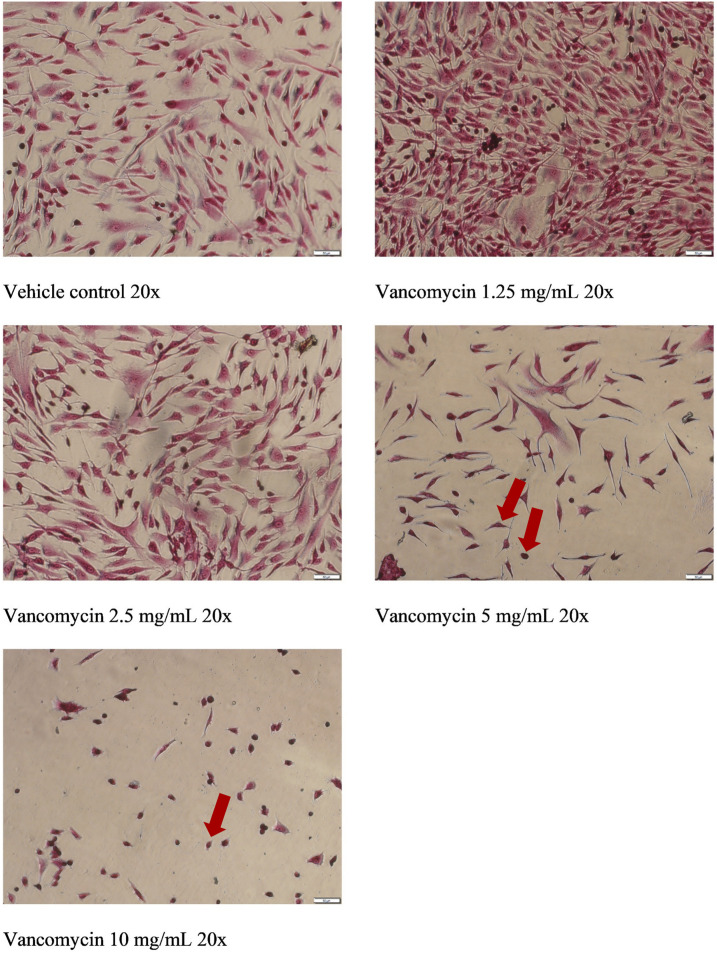
Changes in cellular morphology using crystal violet staining in human primary chondrocytes exposed to vancomycin 1.25 to 10 mg/mL relative to vehicle control. Arrows indicate changes to cell volume and pyknosis.

The morphology of human primary chondrocytes was evaluated using crystal violet staining. The images below (Figure 5) show significant changes in morphology occurred in human primary chondrocytes exposed to various concentrations of vancomycin, in a concentration dependent manner, in comparison to vehicle control. In cells treated with 5 and 10 mg/mL, significantly fewer cells can be seen, consistent with the results observed in the resazurin viability assay. After exposure to both 5 and 10 mg/mL concentrations of vancomycin but to a greater extent 10 mg/mL exposure, chondrocyte cells showed significant morphological changes that included decreased cell volume resulting in cells that became more spherical in shape along with apparent pyknosis, both indicated by arrows in Figure 5.

## Discussion

The aim of the current study was to evaluate the toxicity and underlying mechanisms of vancomycin to knee articular chondrocytes in vitro. This study found that high concentrations of vancomycin (5 and 10 mg/mL) were toxic towards human primary knee chondrocyte cells via apoptotic pathways that may be independent of ROS formation, whilst lower concentrations of vancomycin (1.25 and 2.5 mg/mL) were not toxic.

The prophylactic use of vancomycin in orthopaedic surgeries has become a routine occurrence. Dependent on the surgery, different delivery methods are used for the application of vancomycin to the affected joint with different risks of chondrocyte exposure. A number of studies have previously evaluated the toxicity of vancomycin towards chondrocyte cells in various models, all showing a concentration-dependent toxicity.^11,20^ The current study found that higher concentrations of vancomycin, 5 and 10 mg/mL, were toxic towards NHAC-Kn chondrocytes, as shown by the resazurin reduction assay. This toxicity was shown to be concentration-dependent with highest cell death observed at 10 mg/mL. This is consistent with previous studies showing vancomycin-induced toxicity when chondrocytes were exposed to both 5 and 10 mg/mL concentrations.^9,20^ Hence it is important for clinicians to consider tissue toxicity when topical vancomycin is used during surgery, as certain clinical situations pose greater risk of toxicity than others. For example, infected joint replacements would be considered a low-risk situation as vancomycin powder is typically added to the bone cement.^5,9^ Whilst this vancomycin is usually at extremely high concentrations, chondrocyte toxicity is not a clinical concern as the joint’s articular cartilage has already been removed as part of the joint replacement process. Conversely, using vancomycin to prevent infection secondary to reconstruction of the anterior cruciate ligament in the knee is a higher-risk situation as the native joint is at risk of vancomycin toxicity, hence clinicians need to be aware of this risk if incorrectly undertaken. The technique of Vertullo et al^10^ avoids chondrocyte toxicity by soaking the hamstring graft prior to insertion in a concentration of vancomycin of 5 mg/mL resulting in estimated chondrocyte exposure of 45 to 62 μg/mL to vancomycin, much lower than after direct joint application.^2^ The graft is then rinsed to remove residual vancomycin solution prior to insertion, with the vancomycin eluting into the joint over the next few hours at much lower concentrations.^2^ It should be noted that the vancomycin concentration of 5 mg/mL that causes chondrocyte toxicity is the same concentration required to eliminate *Staphylococcus aureus* from grafts in animal studies.^21^ However, this concentration doesn’t appear to cause graft damage in in vitro cell culture and ex vivo tissue experiments.^22^ Further in vivo studies are required investigating the mechanism of vancomycin toxicity across different concentrations.

Previous studies of vancomycin toxicity have not investigated the cell-death mechanisms causing vancomycin-induced toxicity in chondrocyte cells. In the current study, the effects of ROS production and apoptosis were investigated as possible mechanisms of vancomycin-induced cell death. When evaluated, ROS production in chondrocytes exposed to all concentrations of vancomycin were significantly lower when compared to vehicle control when normalised to cell number although this reduction is to a small degree. This is suggestive that the cell death observed in the current study is independent of ROS production and other cell death mechanisms are likely to be causing the observed vancomycin-induced toxicity although further studies are needed to confirm this. This is despite vancomycin having the ability to induce ROS in renal tubular cells^23^ and may indicate that antioxidant defenses are providing protection in chondrocyte cells, a well-known protective measure in these cells.^24,25^ This is thought to be the first study investigating this effect in chondrocytes, suggesting that vancomycin’s ability to stimulate ROS production may be cell type specific, a well-known phenomenon however, further studies investigating the role of antioxidant defenses is recommended.

The current study showed that caspase-3, an early marker of apoptosis, was activated in chondrocytes treated with 5 and 10 mg/mL suggesting that apoptosis was responsible for the cell death observed. In further support of this, the mitochondrial membrane potential of chondrocytes treated with 10 mg/mL of vancomycin was significantly decreased suggesting membrane instability and apoptosis of the cell. Although not statistically significant, a decrease in the membrane potential of chondrocytes treated with 5 mg/mL of vancomycin was observed. To the best of the authors’ knowledge, this is the first time that this effect has been reported.

Finally, when the morphology of chondrocytes exposed to varying concentrations of vancomycin were assessed, a significant change in the morphology and of the cells was observed at 10 mg/mL and a decrease in the number of cells at 5 mg/mL. This decrease in cell number and the change in morphology is consistent with a decrease in viability of the cells and apoptosis at high concentrations of vancomycin. Other observed morphological changes to the chondrocyte cells showed a decreased cell volume and pyknosis after exposure of these cells to higher concentrations of vancomycin. Both of these morphological changes are indicative that apoptosis has occurred,^26^ providing further evidence to suggest that this pathway is involved in chondrocyte toxicity.

## Conclusion

This study provides further evidence of vancomycin toxicity towards human chondrocytes at high concentrations (≥5 mg/mL). Furthermore, this study provides preliminary evidence that suggests that this toxicity is mediated through apoptotic pathways although further studies are needed to confirm the role of ROS in this process. Clinicians are advised to avoid direct chondrocyte exposure to vancomycin at concentrations of 5 mg/mL or higher. Further studies investigating these and other mechanisms, such as necrosis and autophagy, are needed, especially in regard to additional evidence to support the lack of ROS production, along with in vivo studies to fully elucidate the mechanism of toxicity of vancomycin towards chondrocytes.
